# Cognitive dysfunction associated with cellular immunotherapy for cancer

**DOI:** 10.1093/noajnl/vdag021

**Published:** 2026-03-19

**Authors:** Anna G Hauswirth, Michael W Parsons, Jorg Dietrich

**Affiliations:** Department of Neurology, Division of Neuro-Oncology, Massachusetts General Hospital Cancer Center, Harvard Medical School, Boston, MA, USA; Department of Psychiatry, Massachusetts General Hospital, Harvard Medical School, Boston, MA, USA; Department of Neurology, Division of Neuro-Oncology, Massachusetts General Hospital Cancer Center, Harvard Medical School, Boston, MA, USA

**Keywords:** CAR-T, cognition, cognitive function, ICANS, immunotherapy

## Abstract

Immunotherapies engage the individual’s immune system to fight both hematologic and solid malignancies and have led to durable clinical responses for many patients. A subset of immunotherapies, including T cell engagers (TCEs) and chimeric antigen receptor T-cell therapies (CAR-T), use T cell mediated mechanisms to induce cancer cell death. These therapies can be very effective against the cancer while also causing significant systemic and neurologic toxicity, including cognitive dysfunction. Here, we provide a review on the existing and emerging literature describing the acute, subacute, and potential long-term effects of CAR-T cells and other T-cell-engaging therapies on cognition. While the acute effects on cognition, including immune effector cell-associated neurotoxicity syndrome are becoming better characterized, there is mixed data on the long-term subjective and objective cognitive outcomes after T-cell-based immunotherapies. Further research is needed, especially as these promising and effective therapies become more commonly used for both hematologic and solid malignancies.

Key Points

- Immunotherapies including CAR-T and TCE can cause *acute* cognitive dysfunction including ICANS and as part of tumor inflammation associated neurotoxicity (TIAN; a unique on-target and on-tumor neurotoxicity encountered after CAR-T cell therapies targeting CNS malignancies).
- Less is known about subacute and chronic cognitive effects from immunotherapies, as currently available clinical data is mixed. It remains controversial if and to what extent patients may experience long-term subjective and objective cognitive dysfunction.

Immunotherapies have revolutionized oncology, improving outcomes for many patients with both hematologic and solid tumors. Immunotherapies use a variety of distinct mechanisms to engage the individual’s immune system in fighting cancer. Types of immunotherapies include personalized vaccines, TCEs, checkpoint inhibitors, and cellular therapies, such as chimeric antigen receptor T-cell therapies (CAR-T).^1^

While cell-based immunotherapies are effective for many patients in treating cancer, they are associated with a high frequency of systemic toxicities affecting multiple organ systems, including the central nervous system (CNS).^2^ Neurological symptoms termed immune effector cell-associated neurotoxicity syndrome (ICANS) classically develop after cytokine release syndrome (CRS) and evolve within hours to a few days; with supportive therapy they are usually fully reversible within days. Other neurologic symptoms may have a delayed onset by weeks and can present as movement and cognitive disorders, cranial nerve palsies, and peripheral neuropathies, which are termed non-ICANS neurologic toxicities.^3^^,^^4^

As applications of cellular therapy have expanded and outcomes have improved, studies of the subacute and chronic effects of immunotherapies on cognitive functions—so critically relevant to quality of life for those treated with CAR-T^5^^,^^6^—have gradually proliferated. Early reviews of cognitive outcomes in these patients demonstrated that the literature was limited by heterogeneity among disease groups, treatments, and assessment methods, and had little information about cognitive outcomes beyond the acute period.^7^

Here, we define acute cognitive changes as occurring within 2 weeks of therapy, subacute changes as occurring between 2 weeks and 6 months of therapy, and chronic changes as occurring or persisting after 6 months of therapy.

## Brief Overview of T-cell–Based Therapies

Both TCEs and CAR therapies utilize molecules engineered to target specific cancer antigens and subsequently activate the T cell-mediated immune system to induce cytotoxicity. Many such therapies are in use or in development.

At their simplest design, TCEs bind a cancer associated antigen on 1 arm and a T cell in the other arm (often via the T-cell receptor CD3); when the TCE simultaneously binds the antigen and the T cell, the T cell is activated leading to the desired T-cell-mediated cytotoxicity.^8^^,^^9^ Multiple TCEs have been approved, including for treatment of acute lymphoblastic leukemia, myeloma, melanoma, and small cell lung cancer.^10–14^ Several other TCEs are currently in development, including for primary brain tumors such as glioblastoma.^15^^,^^16^ Additionally, there is ongoing research for their use in other diseases beyond cancers.^17^^,^^18^

CAR therapies utilize genetically engineered T cells with synthetic receptors designed to target specific antigens.^19^ These engineered T cells proliferate in the treated patient and ultimately lead to T-cell-mediated cytotoxicity of the cancer cells. Prior to treatment with CARs, patients typically undergo lymphodepleting chemotherapy with fludarabine and cyclophosphamide to enhance effectiveness of tumor-cell kill by promoting survival of the CARs through depletion of native T cells.^20^ The first CAR-T therapies were developed to target CD19 for treatment of B-cell acute lymphoblastic leukemia and B-cell lymphomas, with further treatments targeting B-cell maturation antigen (BCMA) for treatment of myeloma.^21–26^ There is ongoing work investigating CAR-T therapies for solid tumors, including glioblastoma,^27^^,^^28^ along with autoimmune diseases affecting the nervous system, such as multiple sclerosis, systemic lupus erythematous, and myasthenia gravis.^29^^,^^30^

## Cognitive Assessment of Patients Undergoing T-cell–Based Therapies

Cognition is a fundamental but complex set of neurologic functions that allow an individual to sense, perceive, interpret, plan, and act in their environment.^31^ Both objective and self-reported (eg, subjective) cognitive function measurements are relevant to outcome, and may not correlate.^32^^,^^33^ Strategies to assess *objective* cognitive performance include screening tests like Montreal Cognitive Assessment (MOCA) or Mini-Mental State Examination,^34^ structured neurobehavioral exams such as the immune effector cell-associated encephalopathy (ICE) score,^35^ and formal neuropsychological evaluation. S*ubjective* cognitive surveys, such as the Patient Reported Outcome Measurement Information System Cognitive Function Survey (PROMIS-Cog) and the Functional Assessment of Cancer Therapy-Cognition (FACT-Cog)^34^^,^^36^^,^^37^ provide complementary information. Guidelines for cognitive assessment in the clinical setting (eg, from the National Comprehensive Cancer Network [NCCN]^38^^,^^39^ and the International Partnership for Action Against Cancer [IPAAC])^40^ and in research studies (from the international Cognition and Cancer Task Force [ICCTF])^41^ emphasize the importance of combining both objective and subjective methods of assessment. Ultimately, the goal of cognitive assessment for patients with cancer undergoing complex regimes such as immunotherapy is to balance sensitivity to the domains of interest with efficiency of administration.^33^^,^^41^

## Acute Cognitive Dysfunction After T-cell–Based Therapies

### Clinical Presentation

Patients undergoing treatment with CAR therapies and TCEs are at risk for acute cognitive and neurologic dysfunction within days after treatment. Patients may experience a strong systemic inflammatory response characterized as CRS, characterized by fevers, elevated inflammatory markers, and cytopenias.^42^ Acute to subacute neurotoxicity is also common, with patients often developing a distinct pattern of encephalopathy with confusion, disorientation, aphasia, and impaired fine motor function which is termed ICANS.^3^ The main cognitive domains affected in ICANS are executive functioning, attention, and language.^2^ Patients are also at risk of cerebral edema and seizures, with most severe cases having fatal outcomes.^2^^,^^43^

To monitor for ICANS, patients receiving CAR-T and TCE therapies will undergo regular neurologic assessment via the immune effector cell associated encephalopathy (ICE) score, a simple bedside scale from 1 to 10 that includes tests for attention, language (expressive, receptive, and writing) and orientation.^35^ ICANS classically develops within $$5-10 days of treatment with CAR or TCEs.^10^^,^^44^ Of note, TCE treatments are repeated over time, and development of ICANS often leads to a dose interruption, dose reduction, or discontinuation of the treatment.^10^ Further research is needed to determine if development of ICANS with the first cycle of TCE therapy is associated with increased risk in subsequent cycles; conversely, it is not yet known if absence of ICANS in the first cycle is associated with lower risk of ICANS in future cycles.

Workup for patients with ICANS usually demonstrates elevated systemic inflammatory markers^45^ while cerebrospinal fluid sampling can be bland or show mild inflammatory pleocytosis or elevated protein.^45^ Electroencephalograms (EEGs) can be helpful for patient management, often showing electrical slowing, generalized period discharges (GPDs) or seizures^44^^,^^46^^,^^47^; a recent study also found frontal intermittent rhythmic delta activity (FRIDA) on EEG to be a highly sensitive as a marker for ICANS.^48^ Magnetic resonance imaging (MRI) is often obtained to exclude other causes of neurologic deterioration. Brain MRI in patients affected by ICANS is commonly normal but in some cases varying may abnormalities have been reported, including T2 hyperintensities, hemorrhage or microhemorrhage, diffusion restriction, and contrast enhancement in multiple areas of the brain including the corpus callosum, thalami, basal ganglia, and brainstem.^49^ Transcranial doppler ultrasound can show increased velocities.^50^ With supportive treatment, as further discussed below, symptoms often resolve in days.

CAR-T and TCEs targeting tumors in the brain including lymphoma, glioblastoma and diffuse midline glioma have been associated with neurologic symptoms termed tumor inflammation associated neurotoxicity (TIAN).^51^ TIAN is considered an on-target and on-tumor neurotoxicity syndrome characterized by local inflammation at the site of the tumor and classically presents with worsening of ­previous neurologic deficits. Depending on the location of the tumor and severity of the inflammatory response, these focal neurologic deficits may impact cognition; for example, a patient experiencing TIAN with a left temporal tumor may have worsened aphasia.^3^^,^^52^ However, in severe cases, TIAN may cause brain herniation of obstructive hydrocephalus with impaired cognition and consciousness.^51^

### Epidemiology

ICANS is a common toxicity of CAR-T therapies and somewhat less common, but still prevalent, for many TCEs. It occurs in patients treated with T-cell–based immunotherapies, independent of the specific target of the therapy. Rates of ICANS following CAR-T cell therapy were initially reported in the range of 20-80%, with about 5%-10% of patients having severe or grade 3-4 effects.^2^^,^^24^^,^^46^^,^^50^^,^^53–55^ For patients with TCEs, studies suggest that approximately 20% of patients will develop ICANS, with smaller percentages experiencing severe ICANS, though this appears to be more specific to the type of TCE.^4^

TIAN was noted in 100% of patients receiving CAR-T targeted on GD2 for treatment of diffuse midline gliomas, including TIAN grades 3-4^52^ but may be less common in patients treated for CNS Lymphoma with a reported frequency of ∼20%.^56^ Ongoing safety data and rates of TIAN are still being collected for patients with glioblastoma receiving epidermal growth factor receptor (EGFR) targeted CAR-T.^27^^,^^28^

### Risk Factors

Because of how commonly ICANS develops in patients receiving CAR-T and TCE therapies, there has been significant effort to identify patients who are at risk for ICANS, especially grade 3-5 ICANS. ICANS appears to be more common in those with more aggressive and extensive disease at initiation of the therapy.^45^^,^^57^ Molecular risk factors include baseline inflammation which has also been associated with higher rates of ICANS. Specifically, higher levels of lactate dehydrogenase (LDH), C-reactive protein (CRP), procalcitonin, ferritin, and IL-6 and lower levels of platelets are associated with high rates of serious ICANS.^44^^,^^57^^,^^58^ Additionally, those who have CRS are more likely to develop ICANS.^57^^,^^59^ CNS disease does not seem to be an independent risk factor for ICANS for patients with B-cell malignancies^60^ nor does bridging therapy with chemotherapy or systemic radiation.^57^^,^^61^ Untreated brain metastases in small cell lung cancer may be associated with higher rates of acute neurotoxicity for patients treated with tarlatamab, a TCE against delta-like ligand 3 (DLL-3).^62^

## Subacute Cognitive Dysfunction After T-cell–Based Therapies

### Clinical Presentation

Generally, the acute cognitive dysfunction from ICANS resolves within a few days with supportive care and treatment (see below). Subacute cognitive dysfunction, developing between a few weeks to the first few months has also been observed, and specifically in the context of CAR-T cell therapy targeting BCMA for treatment of refractory multiple myeloma. This treatment has been linked to a unique neurocognitive and movement disorder syndrome termed Movement and Neurocognitive Treatment (MNT) emergent adverse events. Symptoms of MNT classically develop within 2-4 weeks, though can also present later^63^ and are characterized by features of Parkinsonism (rigidity, tremor, bradykinesia), and cognitive dysfunction (confusion, recent memory dysfunction, cognitive slowing, and ­inattention).^46^^,^^63–66^ In some of the reported cases, MRI has shown T2 hyperintensities in the basal ganglia, while [^18^F] fluorodeoxyglucose-positron emission tomography (FDG-PET) has revealed hypometabolism in the basal ganglia.^46^^,^^66^ Studies have identified variable CSF findings, with 1 study showing lymphocytic pleocytosis^65^ and another showing bland CSF.^66^ Patients have been found to have elevated circulating peripheral CARs in high levels as well.^66^ In some cases, these symptoms have not resolved even after many months; for example, 1 patient in the original Ciltacabtagene Autoleucel trial who developed MNT was noted to have ongoing grade 2 symptoms at the time of the data cut in 2022, which was 2 years after the final participant joined the study.^67^ Another patient was noted to have persistent movement symptoms at day 198 after treatment.^65^ Of note, more extensive cognitive testing has not been reported for patients with MNT.

### Epidemiology

In the original study (CARTITUDE-1) investigating BCMA-targeted CAR-T ciltacabtagene autoleucel (cilta-cel), MNT was reported in 5% of patients^63^ (5 out of 97). After more than 2 years of follow up, 1 further case was identified 914 days after infusion.^67^ It has also been reported in 2 cases treated with idecabtagene vicleucel via FDA adverse event monitoring.^68^ A retrospective study of 76 patients who received BCMA targeting CAR-T identified 2 who developed MNT.^46^ In the CARTITUDE-4 study, a phase 3 trial, 208 patients received cilta-cel, and only 1 patient developed grade 2 MNT.^69^

### Risk Factors

Because of the low numbers of patients treated with CAR-T therapies targeting solid tumors like glioblastoma and the low numbers of patients who have developed MNT, risk factors for these toxicities have not been clearly identified. In the original CARTITUDE-1 study, the 5 patients who developed MNT were noted to have at least 2 of the following risk factors: high tumor burden, CRS grade 2 or higher, ICANS of any grade, or high levels of CAR-T expansion and persistence.^63^ Emerging data suggest that a rapid increase in absolute lymphocyte counts (ALC) post CAR-T therapy and persistent high levels of circulating CARs may be important risk factors as well.^64^

## Long-term or Delayed Cognitive Dysfunction After T-cell–Based Therapies

### Clinical Presentation

There is much less known about the chronic effects of immunotherapies on cognition. Overall, studies have not identified significant numbers of patients who experience new or worsening cognitive dysfunction long-term, though studies are limited by small sample sizes, high level of attrition with loss to follow up, and multiple confounding variables including disease progression and other oncologic treatments^70^ (Table 1). High rates of attrition are problematic for longitudinal outcome studies because attrition may not be random. It is possible that patients with greater symptom burden, including cognitive dysfunction are more likely to drop out over time thus confounding cognitive outcome results.^71^ When cognition is evaluated with neuropsychological tests, a handful of studies have shown that cognitive performance appears to be stable 6-12 months after CAR-T compared to baseline,^53^^,^^72–74^ on the group level.

**Table 1.** Selection of studies examining cognition on a sub-acute to chronic time course after CAR-T therapy.

Studies with *subjective* cognitive outcome measures						
Reference	Number of subjects and how subjects were recruited	Diseases	Immunotherapy product	Time period studied	Cognitive tests used	Major Findings
Ruark et al.^8^	40 subjects from a phase I/II trial, contacted to fill out a survey (response rate of 76.9%)	ALL (27.5%) NHL (35%), CLL (37.5%); 15% had CNS involvement	CD-19 targeted CAR-T (product not specified)	1-5 years after CAR-T	Subjective cognitive dysfunction with concentrating, word finding, with memory, or with solving problems (yes or no)	15/40 patients reported cognitive difficulties 1-5 years after CAR-T
Wang et al.^0^	60 subjects, who received CAR-T at a single medical center. Response rate not reported	ALL (5%), DLBCL (95%)	Axicabtagene ciloleucel (86.7%), Tisagenlecleucel (13.3%)	0-12 months after CAR-T	Subjective difficulty remembering on scale from 1 to 10, with >7 representing severe difficulty	19 patients had received CAR-T >3 months prior and on average reported minimal difficulty remembering (mean score of 1.1)
Barata et al.^7^	115 patients prospectively recruited getting CAR-T; 86 did 90-day assessment (11 died, 15 withdrew, 6 refused the survey); 70 did 360-day assessment (13 died, 2 withdrew, 4 refused survey)	NHL	Axicabtagene ciloleucel (85%), 13% tiasgenlecleucel (13%), brexucabrtagene autoleucel (2%)	Baseline, 90- and 360 days post CAR-T	Subjective cognition via Everyday cognition (ECog) scale (includes memory, language, visuospatial abilities, planning, organization, and divided attention)	No change in global cognition or any subdomain at day 90. From 90 to 360, global cognition, memory, language, organization, and divided attention worsened, though absolute changes were small. Worse global cognition with higher ICANS grade
Sidana et al.^9^	34 patients prospectively recruited who underwent CAR-T. 34 completed assessment at week 2. 24 completed assessments at 3 months. 15 completed assessments at 6 months (∼40% had death or change in therapy/progression; ∼20-30% did not complete surveys by choice)	Lymphoma (68%), myeloma (24%), leukemia (9%)	Not specified	Baseline, 2 weeks, 1 month, 2 months, 3 months, 4 months, 5 months, 6 months after CAR-T	Subjective cognition with NeuroQoLv2 questionnaire	No change in self-reported cognitive function over 6 months
Delforge et al.	126 subjects prospectively recruited who underwent CAR-T as part of a clinical trial. 114 were included. 70-90% of patients completed assessments until month 6. 60-70% completed assessments through month 12. 10% completed assessments at 18 months.	Multiple myeloma	Idecabtagene vicleucel	Baseline, monthly for 1-6 months, then every 3 months until 24 months	Subjective cognition as part of larger surveys regarding quality of life	Cognitive functioning improved by month 2 and was stably improved from months 2 to 9 on average. 80% of patients had no change or improvement in cognition over the time points.
Akinola et al.^5^	40 patients recruited after CAR-T and 15 caregivers	Hematologic malignancy	Any CAR-T product	2 weeks to 6 months after CAR-T	Subjective cognition using semi-structured interviews	Qualitative analysis showed patients and caregivers noted cognitive dysfunction, especially forgetfulness
Perthus et al.^6^	59 patients prospectively recruited before undergoing CAR-T. 53 completed 3-month survey. 35 completed 6-month survey. 23 completed 12-month survey. Patients did not complete if disease progression or death or if not followed up. Of note, only 70-80% of these patients completed the cognition survey.	Lymphoma	Axicabtagene ciloleucel (62.7%), tiasgenlecleucel (30.5%), brexucabrtagene autoleucel (6.8%)	Baseline, 3, 6, and 12 months after CAR-T	Subjective cognition (FACT-Cog Scale)	No population level changes in subjective cognition. 13-33% of patients reported significant cognitive decline between 3- and 12 months.

Studies with objective cognitive outcome measures						
Reference	Number of subjects and how subjects were recruited	Diseases	Immunotherapy product	Time period studied	Cognitive tests used	Major Findings
Hoogland et al.^3^	106 prospectively recruited patients receiving CAR-T; 84 completed 30-day assessment; 58 completed 90-day assessment; 54 completed 360-day assessment (combination of patients dying, withdrawing from study, and refusing survey)	NHL	Axicabtagene ciloleucel (87%), tiasgenlecleucel (13%)	Baseline, 30-, 90-, and 360 days post CAR-T	Full neuro-psychological battery	Global cognition decreased slightly from baseline to 90 days, then improved from 90 to 360 days. Patients with grade >2 neurotoxicity had worse cognition. Responders to therapy had better cognition at day 360
Shalabi et al.^4^	52 children and young adults; 37 completed neuro-psychological testing but only 18 completed both baseline and day 21-28 testing (reasons not specified)	Relapsed B-cell malignancies	CD19/CD28 CAR-T	Baseline and 21-28 days after CAR-T	Formal neuro-psychological battery (if age >4)	No difference in executive functioning, attention, working memory, or attention at 1 month when compared to baseline (on average). 88% of individuals had stable to increased score; 10% had declining score
**Studies with both subjective and objective cognitive outcome measures**						
**Reference**	**Number of subjects and how subjects were recruited**	**Diseases**	**Immunotherapy product**	**Time period studied**	**Cognitive tests used**	**Major Findings**
Maillet et al.^3^	52 consecutive adult patients treated with CAR-T at a single center. 27 patients underwent repeat testing (1 lost to follow; 18 died; 6 progressed and were excluded)	Relapsed B-cell lymphoma	Axicabtagene ciloleucel (63%), Tisagenlecleucel (27%)	Baseline prior to CAR-T and then 6-12 months after CAR-T	Full neuro-psychological battery and subjective memory difficulty	3 patients (11%) reported memory difficulty after 6 months (decreased from 30% pre-CAR-T) No worsening in formal neuro-psychological testing per patient between baseline and 6-12 months post-CAR-T. No worsening of cognition for 12 patients who had acute neurotoxicity
Ursu et al.^4^	52 prospectively recruited patients getting CAR-T. Follow up at 2 years for 19 (excluded 20 who died and 13 with progression)	Relapsed B-cell lymphoma patients	CD19 CAR-T	Baseline and then 2 years after (if no progression)	Formal neuro-psychological battery and subjective cognition	No difference at 2 years post CAR-T compared to baseline cognition (both subjective and objective)
Barata et al.^2^	106 subjects completed subjective cognition at baseline; 70 completed it at 6 months. 39 underwent baseline neuro-psychological testing; 26 did at 6 months.	NHL (56.6%), CLL (2.8%), HL (0.9%), ALL (0.9%), MM (25.5%), solid tumor (13.2%)	Axicabtagene ciloleucel (10.4%), tisagenlecleucel (24.5%), lisocabtagene maraleucel (12.3%), tisagenlecleucel (24.5%), brexucabrtagene autoleucel (5.7%), BCMA CAR (24.5%), other (22.6%)	Baseline and 6 months after CAR-T	Formal neuro-psychological battery and subjective cognition (FACT-Cog)	No changes in subjective cognition, in overall cognitive performance on objective testing, or by individual domains. At 6 months, 20% of patients reported declined subjective cognition.

Abbreviations: ALL, acute lymphoblastic leukemia; BCMA, B-cell maturation antigen; CLL, chronic lymphocytic leukemia; CNS, central nervous system; DLBCL, diffuse large B-cell lymphoma; MM, multiple myeloma; NHL, non-Hodgkin’s lymphoma.

Studies of subjective cognitive symptoms have shown mixed results. Qualitative analysis of semi-structured interviews have found that patients report subjective issues with cognition and especially memory dysfunction 4-6 months after receiving CARs.^5^^,^^75^ Qualitative analysis of semi-structured interviews of caregivers also found subjective cognitive dysfunction to be a common theme with 47% of caregivers in 1 study noting cognitive dysfunction in their loved ones.^75^ Patients can report subjective changes in cognition after CAR-T therapies long-term, with some patients describing a decrease in subjective cognition over the first year from CAR-T.^76^ A subset of patients (25%-38%) note worsening of their global cognition that persists 1-5 years after treatment.^77^^,^^78^ Other groups have found that subjective cognition is stable over the first 6 months after CAR-T^79^ or even improved.^6^ Patients with higher grades of ICANS in the acute period are more likely to self-report cognitive issues months later.^77^^,^^80^ It is important to note that even in studies where no changes in cognitive performance were demonstrated at a group level, there have been a range of subjective cognitive outcomes, with 33% of patients reporting improvement, 47% reporting stable, and 20% reporting worsening of their cognitive function on subjective measures in 1 study.^53^^,^^72–74^

In the pediatric population, less is known about long-term cognitive effects of cell-based immunotherapy, with 1 study showing no change in cognition 1 month after CAR-T infusion.^54^ Additional, it is important to note that most patients studied for the long-term cognitive effects of immunotherapies have been patients with B-cell malignancies treated with CAR-T, with much less work done on patients with other malignancies and those treated with TCEs. There is need for further research on the objective and subjective long-term cognitive outcomes following CAR-T and TCEs, including in the pediatric, adult, and geriatric populations.

## Mechanisms of Cognitive Dysfunction

### Immune System and Cytokines

The precise mechanisms leading to altered cognition in patients treated with cell-based therapies remain unknown and poorly understood. Cognitive changes associated with ICANS are likely multifactorial in etiology, with a robust and supraphysiological immune response affecting the CNS considered as a major factor. Mouse models of CAR-T have shown cognitive impairment after treatment, affecting attention, short-term memory, and spatial memory.^81^ Mice were found to have elevated inflammatory markers including in the cerebrospinal fluid and heightened microglial reactivity, which in turn has been shown to affect neuroglial function, myelin integrity and impairs neuronal function and plasticity.^81^ Other studies suggest a role of endothelial dysfunction and blood-brain barrier disruption, with abnormally high levels of inflammatory cytokines and chemokines entering the CNS across a disrupted blood-brain barrier.^19^^,^^44^^,^^45^

### CNS Tumor–Specific Mechanisms

TIAN is generally felt to be due to localized inflammation and edema causing focal neurocognitive and other neurologic symptoms.^51^^,^^56^ Two types of TIAN have been proposed.^51^ Type I has been postulated to originate from localized inflammation and peri-tumoral edema leading to increased intracranial pressure, obstructive hydrocephalus, and potential brain herniation at its most severe form. Type II has been thought to occur from local neuronal dysfunction secondary to an inflammatory response and ultimately resulting in disruption of local circuits. While somnolence and more severe impairment of consciousness can be seen in the setting of type I TIAN as a consequence of elevated intracranial pressure, type II TIAN could drive worsening of language impairment, attention, and executive function depending on the location of the tumor and associated worsening edema. Of note, both types of TIAN can present simultaneously.

### BCMA-Specific Mechanisms

BCMA is not exclusively expressed in hematopoietic cells relevant to the treatment of multiple myeloma. It is also expressed in neurons and astroglia, though there is conflicting data on the level of expression in human brain, especially in the adult brain. A key area of expression is in astroglia and neurons of the basal ganglia, suggesting that MNT as a delayed neurological complication from BCMA-targeted therapy may represent an on-target and off-tumor effect of these cellular immunotherapies.^3^^,^^66^^,^^82^^,^^83^

### Other Contributing Factors for Cognitive Impairment

Of note, patients undergoing cellular immunotherapies often have had many other prior lines of treatments for their cancer, including cytotoxic chemotherapy, stem cell transplantation and lymphodepleting chemotherapy as a conditioning regimen prior to CAR-T cells. In studies of patients undergoing cellular immunotherapies, up to 20% of patients demonstrate cognitive impairment on performance-based measures at the time of immunotherapy and ∼25% report subjective cognitive dysfunction,^72^ likely reflecting the combined effects of other neurotoxic factors, including the neurocognitive effects of the disease and prior treatments.^70^^,^^84^ In this context, the use of fludarabine, known to be associated with subacute and delayed myelin damage and leukoencephalopathy, is an important consideration in the pathophysiology of delayed and chronic cognitive impairment.^85^ Additionally, patients are at risk for infection, ischemic strokes, cerebral hemorrhage, CNS disease, disease progression, seizure, or pre-existing or undiagnosed neurodegenerative disorders. These other conditions can be contributing factors to alterations in cognition, and they could have a confounding or additive effect on top of the cellular therapies themselves.

## Management Options

### Acute/Subacute Cognitive Dysfunction Including ICANS

The backbone of treatment for patients who develop acute to subacute cognitive dysfunction after immunotherapy is supportive care and close monitoring for neurologic deterioration, potentially requiring management in the intensive care unit setting. We would like to refer here to other recent publications which have extensively reviewed the treatment for ICANS, including refractory ICANS.^3^^,^^42^^,^^86^ In brief, moderate to severe cases are usually successfully treated with corticosteroids and other immunomodulatory agents, with refractory cases sometimes treated with systemic and intrathecal chemotherapy and ongoing research on adapter and kill switches to inactivate CARs when needed.^87–92^

### MNT

There are currently no established treatment guidelines for the management of MNT’s and treatment is usually individualized and guided by the treating physician. Typically, similar approaches as for ICANS are employed, with corticosteroids and anakinra as first line intervention^46^ followed by chemotherapy to kill CAR-T cells including intrathecal methotrexate, cytarabine^65^ and cyclophosphamide.^3^^,^^64^ Empiric treatment with levodopa/carbidopa has unfortunately not been effective in most reported cases to treatment of delayed onset Parkinsonism.^66^

### TIAN

Because the mechanism for cognitive impairment from immunotherapies for CNS tumors is felt to be on-target/on-tumor effect, management involves aggressive supportive care and immune modulation as needed.^51^^,^^52^ With optimal supportive care, TIAN is often fully reversible, similar to the reversibility of ICANS.^3^^,^^56^

### Multidisciplinary Treatment of Cognitive Dysfunction

As detailed above, the extent of chronic cognitive dysfunction experienced by patients with cancer that can be attributed to CAR T-cell therapies is an ongoing area of investigation. It is likely that multiple factors contribute to these symptoms, as patients are typically extensively treated with a wide range of regimens than can contribute to cancer-related cognitive impairment (CRCI).^72^ Management of CRCI requires a multidisciplinary team, including cognitive rehabilitation services (including occupational and speech therapy), neuropsychology, and oncology.^93^ Identification and treatment of other causes of cognitive impairment is essential. With increasing knowledge about the pattern and frequency of potential long-term cognitive alterations post CAR-T cells, there is hope that novel interventions can be developed.

### Prevention of Cognitive Dysfunction

Prevention of cognitive dysfunction has historically been based on preventing ICANS. After the original CARTITUDE-1 study identified ∼5% of patients with MNT, the clinical trial team implemented changes to try to prevent and identify MNT early, including reducing tumor burden prior to CAR-T and aggressively treating CRS and ICANS. Lower rates of MNT were observed after these strategies were implemented,^63^^,^^69^ suggesting that such efforts are beneficial in preventing and managing the toxicity.

Regarding other methods used to try to prevent cognitive dysfunction, prophylactic anakinra in a phase 2 study was safe, with suggestion of lower rates of high-grade CRS and ICANS compared to historical data.^94^ Similar findings were seen with prophylactic use of corticosteroids.^95^ There are multiple studies investigating the use of immune modulatory therapy prophylactically to prevent development of ICANS.^3^

## Outlook

CAR-T cells and TCEs are highly effective immunotherapies with the potential to treat a wide range of oncologic conditions, but they carry a risk of cognitive toxicity. Management of acute and subacute neurocognitive syndromes has improved substantially in recent years. However, the extent and frequency of long-term cognitive effects associated with cellular immunotherapies remain poorly defined, underscoring the need for further research.

Emerging evidence suggests that integrating longitudinal subjective and objective cognitive assessments, alongside other patient-centered outcome measures, in larger patient cohorts will be critical to determining whether cell-based therapies lead to persistent cognitive consequences in individual patients. Combining these approaches with conventional and advanced neuroimaging techniques may further elucidate the mechanisms and functional impact of toxicity on neural network systems.

Identifying the precise risk factors associated with cognitive changes will be essential to improving outcomes and developing strategies to mitigate or prevent neurotoxicity. As current cellular immunotherapies continue to be refined and newer, potentially safer CAR-T designs emerge, sustained attention to cognitive outcomes will remain vital to optimizing both clinical efficacy and patients’ quality of life.

## References

[1] Waldman AD , Fritz JM, Lenardo MJ. A guide to cancer immunotherapy: from T cell basic science to clinical practice. Nat Rev Immunol. 2020;20:651-668. 10.1038/S41577-020-0306-5. 32433532 PMC7238960

[2] Grant SJ , Grimshaw AA, Silberstein J, et al. Clinical presentation, risk factors, and outcomes of immune effector cell-associated neurotoxicity syndrome following chimeric antigen receptor T cell therapy: a systematic review. Transplant Cell Ther. 2022;28:294-302. 10.1016/J.JTCT.2022.03.006 35288347 PMC9197870

[3] Karschnia P , Dietrich J. Neurological complications of CAR T cell therapy for cancers. Nat Rev Neurol. 2025;21:422-431. 10.1038/S41582-025-01112-8. 40562951

[4] Zaïr ZM , Butterworth G, Shalaby M, Oštarijaš E, Thisthlethwaite F. T-cell engager toxicity in clinical phase trials; a systematic review and meta-analysis. Cancer Treat Rev. 2025;139:102991. 10.1016/j.ctrv.2025.102991 40644745

[5] Cheng R , Scippa K, Locke FL, Snider JT, Jim H. Patient perspectives on health-related quality of life in diffuse large B-Cell lymphoma treated with car T-Cell therapy: a qualitative study. Oncol Ther. 2022;10:123-141. 10.1007/s40487-021-00174-0 34778941 PMC8590924

[6] Delforge M , Shah N, Miguel JSF, et al. Health-related quality of life with idecabtagene vicleucel in relapsed and refractory multiple myeloma. Blood Adv. 2022;6:1309-1318. 10.1182/BLOODADVANCES.2021005913, 34933328 PMC8864645

[7] Kazzi C , Kuznetsova V, Siriratnam P, et al. Cognition following chimeric antigen receptor T-cell therapy: a systematic review. J Autoimmun. 2023;140:103126. 10.1016/J.JAUT.2023.103126 37837807

[8] Goebeler ME , Bargou RC. T cell-engaging therapies–BiTEs and beyond. Nat Rev Clin Oncol. 2020;17:418-434. 10.1038/s41571-020-0347-5 32242094

[9] Mungalov RV , Mushenkova NV, Chudakov DM, Turchaninova MA. Engaging T cells for cleanup. Front Immunol. 2025;16:1551424. 10.3389/FIMMU.2025.1551424/XML 40416957 PMC12099299

[10] Ahn MJ , Cho BC, Felip E, DeLLphi-301 Investigators, et al. Tarlatamab for patients with previously treated small-cell lung cancer. N Engl J Med. 2023;389:2063-2075. 10.1056/NEJMoa2307980 37861218

[11] Chari A , Minnema MC, Berdeja JG, et al. Talquetamab, a T-Cell–redirecting GPRC5D bispecific antibody for multiple myeloma. N Engl J Med. 2022;387:2232-2244. 10.1056/NEJMoa2204591 36507686

[12] Kantarjian H , Stein A, Gökbuget N, et al. Blinatumomab versus chemotherapy for advanced acute lymphoblastic leukemia. N Engl J Med. 2017;376:836-847. 10.1056/NEJMoa1609783 28249141 PMC5881572

[13] Moreau P , Garfall AL, van de Donk NWCJ, et al. Teclistamab in relapsed or refractory multiple myeloma. N Engl J Med. 2022;387:495-505. 10.1056/NEJMoa2203478 35661166 PMC10587778

[14] Nathan P , Hassel JC, Rutkowski P, IMCgp100-202 Investigators, et al. Overall survival benefit with tebentafusp in metastatic uveal melanoma. N Engl J Med. 2021;385:1196-1206. 10.1056/NEJMoa2103485 34551229

[15] Sternjak A , Lee F, Thomas O, et al. Preclinical assessment of AMG 596, a bispecific T-cell engager (BiTE) immunotherapy targeting the tumor-specific antigen EGFRvIII. Mol Cancer Ther. 2021;20:925-933. 10.1158/1535-7163.MCT-20-0508 33632870

[16] Rosenthal M , Balana C, Linde MV, et al. Novel anti-EGFRvIII bispecific T cell engager (BiTE) antibody construct in glioblastoma (GBM): trial in progress of AMG 596 in patients with recurrent or newly diagnosed disease. JCO. 2019;37:TPS2071-TPS2071. 10.1200/JCO.2019.37.15_SUPPL.TPS2071

[17] Hagen M , Bucci L, Böltz S, et al. BCMA-targeted T-cell–engager therapy for autoimmune disease. N Engl J Med. 2024;391:867-869. 10.1056/NEJMC2408786 39231353

[18] Bucci L , Hagen M, Rothe T, et al. Bispecific T cell engager therapy for refractory rheumatoid arthritis. Nat Med. 2024;30:1593-1601. 10.1038/S41591-024-02964-1 38671240

[19] June CH , Sadelain M. Chimeric antigen receptor therapy. N Engl J Med. 2018;379:64-73. 10.1056/NEJMRA1706169 29972754 PMC7433347

[20] Lickefett B , Chu L, Ortiz-Maldonado V, et al. Lymphodepletion–an essential but undervalued part of the chimeric antigen receptor T-cell therapy cycle. Front Immunol. 2023;14:1303935. 10.3389/FIMMU.2023.1303935 38187393 PMC10770848

[21] Gardner RA , Finney O, Annesley C, et al. Intent-to-treat leukemia remission by CD19 CAR T cells of defined formulation and dose in children and young adults. Blood. 2017;129:3322-3331. 10.1182/BLOOD-2017-02-769208 28408462 PMC5482103

[22] Maude SL , Laetsch TW, Buechner J, et al. Tisagenlecleucel in children and young adults with B-Cell lymphoblastic leukemia. N Engl J Med. 2018;378:439-448. 10.1056/NEJMOA1709866 29385370 PMC5996391

[23] Neelapu SS , Locke FL, Bartlett NL, et al. Axicabtagene ciloleucel CAR T-Cell therapy in refractory large B-Cell lymphoma. N Engl J Med. 2017;377:2531-2544. doi:10.1056/NEJMOA1707447 29226797 10.1056/NEJMoa1707447 PMC5882485

[24] Schuster SJ , Bishop MR, Tam CS, JULIET Investigators, et al. Tisagenlecleucel in adult relapsed or refractory diffuse large B-Cell lymphoma. N Engl J Med. 2019;380:45-56. 10.1056/NEJMOA1804980 30501490

[25] Berdeja JG , Madduri D, Usmani SZ, et al. Ciltacabtagene autoleucel, a B-cell maturation antigen-directed chimeric antigen receptor T-cell therapy in patients with relapsed or refractory multiple myeloma (CARTITUDE-1): a phase 1b/2 open-label study. *The.* Lancet. 2021;398:314-324. 10.1016/S0140-6736(21)00933-8 34175021

[26] Raje N , Berdeja J, Lin Y, et al. Anti-BCMA CAR T-Cell therapy bb2121 in relapsed or refractory multiple myeloma. N Engl J Med. 2019;380:1726-1737. 10.1056/NEJMOA1817226 31042825 PMC8202968

[27] Bagley SJ , Logun M, Fraietta JA, et al. Intrathecal bivalent CAR T cells targeting EGFR and IL13Rα2 in recurrent glioblastoma: phase 1 trial interim results. Nat Med. 2024;30:1320-1329. 10.1038/S41591-024-02893-Z 38480922 PMC13123313

[28] Choi BD , Gerstner ER, Frigault MJ, et al. Intraventricular CARv3-TEAM-E T cells in recurrent glioblastoma. N Engl J Med. 2024;390:1290-1298. 10.1056/NEJMOA2314390 38477966 PMC11162836

[29] Müller F , Taubmann J, Bucci L, et al. CD19 CAR T-Cell therapy in autoimmune disease–a case series with follow-up. N Engl J Med. 2024;390:687-700. 10.1056/NEJMOA2308917 38381673

[30] Yang C , Sun C, Tan B, et al. Allogeneic anti-CD19 CAR-T cells induce remission in refractory systemic lupus erythematosus. Cell Res. 2025;35:607-609. 10.1038/S41422-025-01128-1;TECHMETA 40341741 PMC12297247

[31] Harvey PD. Domains of cognition and their assessment. Dialogues Clin Neurosci. 2019;21 3 227-237. 10.31887/DCNS.2019.21.3/PHARVEY, 31749647 PMC6829170

[32] Sachdev PS , Blacker D, Blazer DG, et al. Classifying neurocognitive disorders: the DSM-5 approach. Nat Rev Neurol. 2014;10:634-642. 10.1038/NRNEUROL.2014.181 25266297

[33] Costa DSJ , Fardell JE. Why are objective and perceived cognitive function weakly correlated in patients with cancer? J Clin Oncol. 2019;37:1154-1158. 10.1200/JCO.18.02363 30920881

[34] Rambeau A , Beauplet B, Laviec H, et al. Prospective comparison of the montreal cognitive assessment (MoCA) and the mini mental state examination (MMSE) in geriatric oncology. J Geriatr Oncol. 2019;10:235-240. 10.1016/J.JGO.2018.08.003 30150019

[35] Lee DW , Santomasso BD, Locke FL, et al. ASTCT consensus grading for cytokine release syndrome and neurologic toxicity associated with immune effector cells. Biol Blood Marrow Transplant. 2019;25:625-638. 10.1016/J.BBMT.2018.12.758 30592986 PMC12180426

[36] Cella D , Choi S, Garcia S, et al. Setting standards for severity of common symptoms in oncology using the PROMIS item banks and expert judgment. Qual Life Res. 2014;23:2651-2661. 10.1007/S11136-014-0732-6 24938431 PMC4710358

[37] Iverson GL , Marsh JM, Connors EJ, Terry DP. Normative reference values, reliability, and Item-level symptom endorsement for the PROMIS^®^ v2.0 cognitive Function-Short forms 4a, 6a and 8a. Arch Clin Neuropsychol. 2021;36 7 1341-1349. 10.1093/ARCLIN/ACAA128 33454756

[38] Ansbaugh SM , Advocate Ella Ariza-Heredia PJ, Scott Baker K, et al. NCCN Guidelines Version 2.2025 Survivorship. Published online 2025. https://www.nccn.org/guidelines/guidelines-detail?category=3&id=1466

[39] Sanft T , Day AT, Ansbaugh SM, et al. NCCN guidelines^®^ insights: Survivorship, version 2.2025: featured updates to the NCCN guidelines^®^. J Natl Compr Canc Netw. 2025;23:208-217. 10.6004/JNCCN.2025.0028 40499584

[40] iPAAC. Guide: Practices and recommendations for the management of cognitive impairments after cancer. Published online 2021. Accessed September 23, 2025. https://www.ipaac.eu/res/file/outputs/wp4/practices-recommendations-cancer-cognitive-impairements.pdf

[41] Wefel JS , Vardy J, Ahles T, Schagen SB. International cognition and cancer task force recommendations to harmonise studies of cognitive function in patients with cancer. Lancet Oncol. 2011;12:703-708. 10.1016/S1470-2045(10)70294-1 21354373

[42] Jain MD , Smith M, Shah NN. How I treat refractory CRS and ICANS after CAR T-cell therapy. Blood. 2023;141:2430-2442. 10.1182/BLOOD.2022017414 36989488 PMC10329191

[43] Gutierrez C , Brown ART, May HP, et al. Critically ill patients treated for chimeric antigen receptor related toxicity: a multicenter study. Crit Care Med. 2022;50 1 81-92. 10.1097/CCM.0000000000005149 34259446 PMC8678137

[44] Karschnia P , Jordan JT, Forst DA, et al. Clinical presentation, management, and biomarkers of neurotoxicity after adoptive immunotherapy with CAR T cells. Blood. 2019;133:2212-2221. 10.1182/BLOOD-2018-12-893396 30808634

[45] Santomasso BD , Park JH, Salloum D, et al. Clinical and biological correlates of neurotoxicity associated with car t-cell therapy in patients with B-cell acute lymphoblastic leukemia. Cancer Discov. 2018;8:958-971. 10.1158/2159-8290.CD-17-1319 29880584 PMC6385599

[46] Karschnia P , Miller KC, Yee AJ, et al. Neurologic toxicities following adoptive immunotherapy with BCMA-directed CAR T cells. Blood. 2023;142:1243-1248. 10.1182/BLOOD.2023020571 37471607

[47] Herlopian A , Dietrich J, Abramson JS, Cole AJ, Westover MB. EEG findings in CAR T-cell therapy-related encephalopathy. Neurology. 2018;91:227-229. 10.1212/WNL.0000000000005910 29959264 PMC6093761

[48] Huby S , Gelisse P, Tudesq JJ, et al. Frontal intermittent rhythmic Delta activity is a useful diagnostic tool of neurotoxicity after CAR T-Cell infusion. Neurol Neuroimmunol Neuroinflamm. 2023;10:e200111. 10.1212/NXI.0000000000200111 37059470 PMC10119810

[49] Vichare A , Lee JS, Duong TQ. Neuroimaging findings of CAR T-cell-­associated neurotoxicity: a review. Neurol Clin Pract. 2025;15:e200488- 10.1212/CPJ.0000000000200488 40687961 PMC12270458

[50] Rubin DB , Danish HH, Ali AB, et al. Neurological toxicities associated with chimeric antigen receptor T-cell therapy. Brain. 2019;142:1334-1348. 10.1093/BRAIN/AWZ053 30891590

[51] Mahdi J , Dietrich J, Straathof K, et al. Tumor inflammation-associated neurotoxicity. Nat Med. 2023;29:803-810. 10.1038/S41591-023-02276-W 37024595 PMC10166099

[52] Monje M , Mahdi J, Majzner R, et al. Intravenous and intracranial GD2-CAR T cells for H3K27M+ diffuse midline gliomas. Nature. 2025;637:708-715. 10.1038/S41586-024-08171-9 39537919 PMC11735388

[53] Maillet D , Belin C, Moroni C, et al. Evaluation of mid-term (6-12 months) neurotoxicity in B-cell lymphoma patients treated with CAR T cells: a prospective cohort study. Neuro Oncol. 2021;23:1569-1575. 10.1093/neuonc/noab077 33822183 PMC8408887

[54] Shalabi H , Martin S, Yates B, et al. Neurotoxicity following CD19/CD28ζ CAR T-cells in children and young adults with B-cell malignancies. Neuro Oncol. 2022;24:1584-1597. 10.1093/NEUONC/NOAC034 35148417 PMC9435493

[55] Abramson JS , Palomba ML, Gordon LI, et al. Lisocabtagene maraleucel for patients with relapsed or refractory large B-cell lymphomas (TRANSCEND NHL 001): a multicentre seamless design study. Lancet. 2020;396:839-852. 10.1016/S0140-6736(20)31366-0 32888407

[56] Kaulen LD , Martinez-Lage M, Abramson JS, et al. Clinical presentation, management, and outcome of TIAN in CNS lymphoma treated with CD19-CAR T-cell therapy. Blood. 2025;146:1902-1913. 10.1182/BLOOD.2025028964 Published online July 15, 40663771

[57] Rubin DB , Al Jarrah A, Li K, et al. Clinical predictors of neurotoxicity after chimeric antigen receptor T-Cell therapy. JAMA Neurol. 2020;77:1536-1542. 10.1001/JAMANEUROL.2020.2703 32777012 PMC7418044

[58] Pennisi M , Sanchez-Escamilla M, Flynn JR, et al. Modified EASIX predicts severe cytokine release syndrome and neurotoxicity after chimeric antigen receptor T cells. Blood Adv. 2021;5:3397-3406. 10.1182/BLOODADVANCES.2020003885 34432870 PMC8525234

[59] Gajra A , Zettler ME, Phillips EG, et al. Neurological adverse events following CAR T-Cell therapy: a Real-World analysis. Immunotherapy. 2020;12:1077-1082. 10.2217/IMT-2020-0161 32808566

[60] Karschnia P , Arrillaga-Romany IC, Eichler A, et al. Neurotoxicity and management of primary and secondary central nervous system lymphoma after adoptive immunotherapy with CD19-directed chimeric antigen receptor T-cells. Neuro Oncol. 2023;25:2239-2249. 10.1093/NEUONC/NOAD118 37402650 PMC10708936

[61] Roddie C , Neill L, Osborne W, et al. Effective bridging therapy can improve CD19 CAR-T outcomes while maintaining safety in patients with large B-cell lymphoma. Blood Adv. 2023;7:2872-2883. 10.1182/BLOODADVANCES.2022009019 36724512 PMC10300297

[62] Bolte FJ , Dougherty SC, Danos AO, et al. Real-world outcomes of tarlatamab in small cell lung cancer, including patients with untreated brain metastases. Clin Lung Cancer. 2025;26:347-353.e1. 10.1016/J.CLLC.2025.03.006 40280845

[63] Cohen AD , Parekh S, Santomasso BD, et al. Incidence and management of CAR-T neurotoxicity in patients with multiple myeloma treated with ciltacabtagene autoleucel in CARTITUDE studies. Blood Cancer J. 2022;12:32- 10.1038/S41408-022-00629-1 35210399 PMC8873238

[64] Graham CE , Lee WH, Wiggin HR, et al. Chemotherapy-induced reversal of ciltacabtagene autoleucel–associated movement and neurocognitive toxicity. Blood. 2023;142:1248-1252. 10.1182/BLOOD.2023021429 37467494 PMC10579042

[65] Gudera JA , Baehring JM, Karschnia P. Parkinsonism following chimeric antigen receptor T cell therapy. JAMA Neurol. 2024;81:1223-1224. 10.1001/jamaneurol.2024.2506 39133506

[66] Van Oekelen O , Aleman A, Upadhyaya B, et al. Neurocognitive and hypokinetic movement disorder with features of parkinsonism after BCMA-targeting CAR-T cell therapy. Nat Med. 2021;27:2099-2103. 10.1038/s41591-021-01564-7 34893771 PMC8678323

[67] Martin T , Usmani SZ, Berdeja JG, et al. Ciltacabtagene autoleucel, an anti-B-cell maturation antigen chimeric antigen receptor T-Cell therapy, for relapsed/refractory multiple myeloma: CARTITUDE-1 2-Year Follow-Up. J Clin Oncol. 2023;41:1265-1274. 10.1200/JCO.22.00842 35658469 PMC9937098

[68] Gong Z , Umoru G, Monge J, et al. Adverse effects and non-relapse mortality of BCMA-directed immunotherapies : an FDA adverse event reporting system (FAERS) database study. Blood. 2023;142:358. 10.1182/BLOOD-2023-182695

[69] San-Miguel J , Dhakal B, Yong K, et al. Cilta-cel or standard care in Lenalidomide-Refractory multiple myeloma. N Engl J Med. 2023;389:335-347. 10.1056/NEJMoa2303379 37272512

[70] Ahles TA , Root JC. Cognitive effects of cancer and cancer treatments. Annu Rev Clin Psychol. 2018;14:425-451. 10.1146/annurev-clinpsy-050817-084903 29345974 PMC9118140

[71] Hui D , Glitza I, Chisholm G, Yennu S, Bruera E. Attrition rates, reasons, and predictive factors in supportive care and palliative oncology clinical trials. Cancer. 2013;119:1098-1105. 10.1002/CNCR.27854 23132290 PMC3568443

[72] Barata A , Johnson PC, Dhawale TM, et al. Long-term cognitive outcomes in adult patients receiving chimeric antigen receptor T-Cell therapies. Transplant Cell Ther. 2025;31:236.e1-236.e13. 10.1016/j.jtct.2025.01.886 PMC12204036 39870307

[73] Hoogland AI , Barata A, Logue J, et al. Change in neurocognitive performance among patients with non-Hodgkin lymphoma in the first year after chimeric antigen receptor T cell therapy. Transplant Cell Ther. 2022;28:305.e1-305-e9. 10.1016/j.jtct.2022.03.023 PMC9197947 35378330

[74] Ursu R , Maillet D, Belin C, et al. Long-term neurologic safety in patients with B-Cell lymphoma treated with anti-CD19 chimeric antigen receptor T-Cell therapy. Neurology. 2022;99:511-515. 10.1212/WNL.0000000000201083, 35851255

[75] Akinola IM , Cusatis R, Pasquini MC, et al. Multi-stakeholder qualitative interviews to inform measurement of patient reported outcomes after CAR-T. Transplant Cell Ther. 2023;29:254.e1-254-e9. 10.1016/J.JTCT.2023.01.004 PMC10369368 36634738

[76] Perthus A , Colin F, Charton E, et al. Remission after CAR T-cell therapy: do lymphoma patients recover a normal life? Hemasphere. 2024;8:e72- 10.1002/HEM3.72 38803454 PMC11129324

[77] Barata A , Hoogland AI, Kommalapati A, et al. Change in patients’ perceived cognition following chimeric antigen receptor T-Cell therapy for lymphoma. Transplant Cell Ther. 2022;28:401.e1-401-e7. 10.1016/j.jtct.2022.05.015 PMC9339228 35580732

[78] Ruark J , Mullane E, Cleary N, et al. Patient-reported neuropsychiatric outcomes of long-term survivors after chimeric antigen receptor T cell therapy. Biol Blood Marrow Transplant. 2020;26:34-43. 10.1016/j.bbmt.2019.09.037 31605820 PMC6951812

[79] Sidana S , Dueck AC, Thanarajasingam G, et al. Longitudinal patient reported outcomes with CAR-T cell therapy versus autologous and allogeneic stem cell transplant. Transplant Cell Ther. 2022;28:473-482. 10.1016/J.JTCT.2022.05.004 35550440 PMC9357185

[80] Wang XS , Srour SA, Whisenant M, et al. Patient-Reported symptom and functioning status during the first 12 months after chimeric antigen receptor T cell therapy for hematologic malignancies. Transplant Cell Ther. 2021;27:930.e1-930.e10. 10.1016/j.jtct.2021.07.007 PMC9710064 34265479

[81] Geraghty AC , Acosta-Alvarez L, Rotiroti MC, et al. Immunotherapy-related cognitive impairment after CAR T cell therapy in mice. bioRxiv. 2025;188:3238-3258.e25. 10.1101/2024.05.14.594163 PMC12176077 40359942

[82] Mohyuddin GR , Banerjee R, Alam Z, Berger KE, Chakraborty R. Rethinking mechanisms of neurotoxicity with BCMA directed therapy. Crit Rev Oncol Hematol. 2021;166:103453- 10.1016/J.CRITREVONC.2021.103453 34461271

[83] Marella M , Yao X, Carreira V, et al. Comprehensive BCMA expression profiling in adult normal human brain suggests a low risk of on-target neurotoxicity in BCMA-targeting multiple myeloma therapy. J Histochem Cytochem. 2022;70 4 273-287. 10.1369/00221554221079579 35193424 PMC8971684

[84] Janelsins MC , Kesler SR, Ahles TA, Morrow GR. Prevalence, mechanisms, and management of cancer-related cognitive impairment. Int Rev Psychiatry. 2014;26:102-113. 10.3109/09540261.2013.864260 24716504 PMC4084673

[85] Winter SF , Martinez-Lage M, Clement NF, Hochberg EP, Dietrich J. Fatal neurotoxicity after chimeric antigen receptor T-cell therapy: an unexpected case of fludarabine-associated progressive leukoencephalopathy. Eur J Cancer. 2021;144:178-181. 10.1016/j.ejca.2020.11.021 33360262

[86] Santomasso BD , Nastoupil LJ, Adkins S, et al. Management of immune-related adverse events in patients treated with chimeric antigen receptor T-Cell therapy: ASCO guideline. J Clin Oncol. 2021;39:3978-3992. 10.1200/JCO.21.01992 34724386

[87] Wehrli M , Gallagher K, Chen YB, et al. Single-center experience using anakinra for steroid-refractory immune effector cell-associated neurotoxicity syndrome (ICANS). J Immunother Cancer. 2022;10:e003847. 10.1136/JITC-2021-003847, PMC8744112 34996813

[88] Strati P , Ahmed S, Kebriaei P, et al. Clinical efficacy of anakinra to mitigate CAR T-cell therapy–associated toxicity in large B-cell lymphoma. Blood Adv. 2020;4:3123-3127. 10.1182/BLOODADVANCES.2020002328 32645136 PMC7362369

[89] Bajwa A , Zhao Q, Geer M, et al. Siltuximab for chimeric antigen receptor T-cell therapy–related CRS and ICANS: a multicenter retrospective ­analysis. Blood Adv. 2025;9:170-175. 10.1182/BLOODADVANCES.2024013688 39437770 PMC11788129

[90] Blumenberg V , Puliafito BR, Graham CE, et al. Cyclophosphamide mitigates non-ICANS neurotoxicities following ciltacabtagene autoleucel treatment. Blood. 2025;145:2788-2793. 10.1182/BLOOD.2024028172, 40163799 PMC12782967

[91] Shah NN , Johnson BD, Fenske TS, Raj RV, Hari P. Intrathecal chemotherapy for management of steroid-refractory CAR T-cell-associated neurotoxicity syndrome. Blood Adv. 2020;4:2119-2122. 10.1182/BLOODADVANCES.2020001626, 32407473 PMC7252557

[92] Lu L , Xie M, Yang B, Zhao W, Bin, Cao J. Enhancing the safety of CAR-T cell therapy: Synthetic genetic switch for spatiotemporal control. Sci Adv. 2024;10:eadj6251- 10.1126/sciadv.adj6251 38394207 PMC10889354

[93] Sleurs C , Reilly J, Santarnecchi E, Parsons MW. Neuropsychological functioning in non-CNS cancer patients. J Clin Exp Neuropsychol. 2025;47:749-767. 10.1080/13803395.2025.2523368; SUBPAGE: STRING: FULL Published online September 14, 40619814

[94] Park JH , Sauter CS, Palomba ML, et al. A phase II study of prophylactic anakinra to prevent CRS and neurotoxicity in patients receiving CD19 CAR T cell therapy for relapsed or refractory lymphoma. Blood. 2021;138:96- 10.1182/BLOOD-2021-150431

[95] Oluwole OO , Bouabdallah K, Muñoz J, et al. Prophylactic corticosteroid use in patients receiving axicabtagene ciloleucel for large B-cell lymphoma. Br J Haematol. 2021;194:690-700. 10.1111/BJH.17527 34296427 PMC8457222

